# Curriculum Innovations: Near-Peer Learning in Neurology Residency Training on Cerebrovascular Disease in a Teaching Hospital in China

**DOI:** 10.1212/NE9.0000000000200072

**Published:** 2023-05-10

**Authors:** Yuehui Hong, Ning Su, Hanhui Fu, Yuze Cao, Ming Yao, Lixin Zhou, Jun Ni, Yicheng Zhu

**Affiliations:** From the Department of Neurology, State Key Laboratory of Complex Severe and Rare Diseases, Peking Union Medical College Hospital, Chinese Academy of Medical Science & Peking Union Medical College, Beijing, China.

## Abstract

**Introduction and Problem Statement:**

Traditional faculty-led training is teacher-centric and requires substantial investments in teaching faculty and resources. Near-peer learning (NPL) is a teaching strategy in which senior residents instruct juniors who are only 1 or 2 years earlier in their training. NPL promotes student engagement and may enhance teaching competency of participants. We implemented an NPL instructional design for a course on cerebrovascular disease for residents in China.

**Objectives:**

Tutors and tutees will be able to (1) demonstrate knowledge of cerebrovascular anatomy, (2) understand cerebrovascular physiology, and (3) use neuroimaging and physiology to evaluate cerebrovascular pathology.

**Methods and Curriculum Description:**

From December 2019 to March 2022, NPL was implemented in a neurology residency training program in China. A series of NPL lectures was conducted in addition to traditional faculty-led lectures. The NPL intervention consisted of senior resident tutors who designed and led lectures on foundational topics in cerebrovascular neurology (e.g., anatomy and imaging of cerebral blood vessels, clinical and imaging features of ischemic and hemorrhagic stroke) under the guidance of faculty instructors. Tutees were junior residents in the same program. Precourse/postcourse examinations and feedbacks through online questionnaires were used to evaluate the effectiveness of the NPL intervention on knowledge acquisition and teaching competency.

**Results and Assessment Data:**

Over 3 academic years, 57 total residents participated, including 18, 18, and 21, respectively. All participated in the NPL intervention, with some attending more than once. Participants could be assigned as tutor or tutee in different years. Eighteen lectures were delivered by 15 tutors. The rest were tutees. Postcourse examination scores improved significantly compared with precourse scores (64.22 ± 12.11 vs 59.80 ± 15.88, *p* = 0.003), with the most remarkable improvements seen for the first-year residents and first-time participants. One hundred sixty-two postsession feedbacks from participants (both tutors and tutees) and 15 postprogram feedbacks from tutors were collected. Respondents thought highly of NPL, reporting gain in knowledge and teaching opportunities.

**Discussion and Lessons Learned:**

NPL enabled residents to acquire foundational knowledge of cerebrovascular diseases and provided senior residents with teaching opportunities.

## Introduction and Problem Statement

During the past decades, tremendous changes have taken place in medicine. There are larger patient volumes, higher expectations, more complicated medical management, and rigorous legal regulations. As a result, residency training worldwide must adapt to the changing landscape of medicine.^1^ The nationwide reform of medical education in China is complicated by its vastness, diversity, and unique history. To set national quality standards, China launched the 5+3 system, which comprises 5 years of undergraduate medical education and 3 years of standardized residency training (SRT), similar to US residency training.^2^ SRT requires trainees to practice under supervision in relevant departments (including neurology, neurosurgery, emergency department, internal medicine, and radiology) to gain qualifications for independent practice. On completion of the 3-year training, residents must pass a 2-step test (clinical skills and clinical knowledge) in accredited training bases to ultimately achieve certification.^2,3^

Neurology residency training is complicated. Many trainees find neuroanatomy and neurophysiology to be complex. Advances in diagnostic and therapeutic technology have made medical care more complex to practice, learn, and master. Reliance on technology may lead to difficulty in synthesizing and understanding underlying pathophysiology, especially for beginners.^1^ Faculty-led training tends to be teacher-centric and lacks student engagement.^4^ Teaching faculty and resources are often insufficient in undeveloped hospitals, which is another major challenge that should be addressed by China's health reform.

Near-peer learning (NPL) is an innovative teaching strategy that was first introduced in the late 1980s by Whitman.^5,6^ During NPL, senior learners teach junior colleagues.^7,8^ Compared with traditional teaching modalities such as faculty-led learning, residents participating in NPL not only learn “twice” but also learn in a different way. They are more likely to adopt a step-by-step approach and think from a beginner's perspective. Psychological safety can be established with an atmosphere that may be perceived as less stressful and facilitate student engagement and increased participation. Therefore, NPL could not only promote more efficient knowledge acquisition but also enhance teaching competency. Well-trained SRT residents could constitute teaching faculty for future medical work forces, such as general practitioners.^2,6,8^ These advantages of NPL make it a potentially plausible part of the current standardized neurology residency training in China.

In this pilot study, we applied NPL to train neurology residents in cerebrovascular disease, one of the most common and challenging disorders in neurology. Cerebrovascular disease is essential for doctors in non-neurological specialties as well. We hypothesized that NPL could promote knowledge acquisition and enhance teaching competency in residency training in China.

## Objectives

By the end of the NPL intervention, both tutors and tutees would be able to (1) demonstrate knowledge of cerebrovascular anatomy, (2) understand cerebrovascular physiology, and (3) use neuroimaging and physiology to evaluate cerebrovascular pathology. In addition, tutors would gain teaching experience.

## Methods and Curriculum Description

### Program Setting

Since 2019, peer-led (NPL) lectures have been integrated into faculty-led (traditional) lectures within the residency training program in the Department of Neurology, Peking Union Medical College Hospital (PUMCH). The NPL intervention consisted of foundational lectures provided by senior residents to near-peer juniors on anatomy, physiology, and imaging. Traditional faculty-led lectures covered more specialized topics such as acute stroke management (e.g., reperfusion therapy in ischemic stroke) and under-recognized cerebrovascular diseases (e.g., stroke in young adults, cerebral venous thrombosis). NPL covers 3 sessions that each included a lecture, followed by case-based discussion. The session topics were (1) anatomy of cerebral blood vessels: shape, course, and segmentation of blood vessels shown by imaging—MR angiography; CT angiography; digital subtraction angiography; and the foundational principles, parameters, and applications of transcranial Doppler (TCD)—and the imaging characteristics of special cerebrovascular diseases (i.e., arterial dissection, aneurysm, arteriovenous malformation, and moyamoya disease/syndrome); (2) ischemic stroke: brain perfusion physiology, clinical and radiologic diagnosis of ischemic stroke, pathophysiologic mechanism of cerebral infarction, and etiologic classification of cerebral infarction (i.e., Trial of Org 10172 in Acute Stroke Treatment and Chinese ischemic stroke subclassification); and (3) hemorrhagic stroke: clinical and imaging features of common hemorrhagic cerebrovascular diseases, anatomy of the cerebral venous system, and clinical and imaging features of cerebral venous thrombosis. NPL sessions were held every 1–2 weeks. Each session was 90 minutes.

Recommended learning materials and resources were (1) Neurology^9^; (2) Duus' Topical Diagnosis in Neurology: Anatomy, Physiology, Signs, Symptoms^10^; (3) Pocket Atlas of Sectional Anatomy^11^; (4) Anatomic Basis of Neurologic Diagnosis^12^; (5) Caplan's Stroke: A Clinical Approach^13^; and (6) radiopaedia.org/.

### Program Description

#### Role Assignments

NPL consisted of 4 roles: tutors, tutees, teaching assistants, and instructors. Tutors were third-year residents who were assigned by the teaching assistant (2 tutors were required for each session; therefore, a total of 6 tutors were required each academic year). Tutees were first and second-year residents and the remaining third-year residents not serving as tutors. Teaching assistants were 2 stroke fellows who were assigned by the instructor each academic year. Instructors were clinicians who specialized in cerebrovascular disease with years of clinical practice and teaching experience.

#### Procedure

The instructors formulated learning objectives and teaching content and recommended reading materials for each session. The teaching assistants collected clinical cases on cerebrovascular diseases and developed step-by-step questions for case discussion. The tutors prepared and led the lectures. They also conceptualized how the cases would be presented and taught. Each tutor gave a mock lecture to the instructors and made adjustments accordingly before lecturing and leading case discussions to the tutees officially. The tutees were divided into 3–4 groups to discuss cases. Each group designated a representative to report on their interpretation of the neuroimage and leading diagnosis to the large group. Tutors offered immediate feedbacks to the tutees, and instructors gave their final comments during the session.

### Program Assessment

#### Examination

Knowledge acquisition (level 2 of the Kirkpatrick model) was assessed through precourse and postcourse examinations. Every examination consisted of 10 open-ended questions, with 10 points marked for each. The questions were based on 10 stroke cases with typical presentations and common etiologies (including ischemic stroke caused by large atherosclerosis; small artery occlusion; branch artery disease; cardiac embolism; artery dissection; and hemorrhagic stroke caused by hypertension, amyloid angiography, and aneurysm). The clinical information and neuroimaging were presented, and the residents were asked to interpret the image and provide the most likely diagnosis (including the stroke etiology). All the questions were chosen from the neurology examination database with similar difficulty levels. The database was made by 5 expert teaching faculty with years of experience in clinical neurology practice. It consisted of questions from national and institutionally generated examinations. The questions were vetted by agreement among the 5 experts. The instructors (J.N. and L.Z.) revised the examination and approved the final version. Both of the instructors had received a teacher certification granted by the Ministry of Education of the People's Republic of China. The examinations were taken by all residents (both tutors and tutees) within 30 minutes before the first session and after all sessions were completed. Examinations were later evaluated by the teaching assistants and instructors who were blinded to participants.

#### Feedback

Anonymous questionnaires evaluated learner perceptions (level 1 of the Kirkpatrick model) after each session. Surveys were formulated by the instructors (J.N. and L.Z.) and teaching assistants (Y.H., N.S., H.F., and Y.C.). They were distributed online using the Wenjuanxing platform (wjx.cn). Questionnaires consisted of postsession feedback and postprogram feedback. Postsession feedback was collected from all participants (i.e., residents, fellows, and visiting doctors) immediately after each session. Postprogram feedback was completed by tutors after 1–3 years of the course to evaluate the long-term effect of NPL on tutors. Questionnaires contained 1 open-ended question (“Do you have any suggestions for integrating NPL in neurology residency training?”), and 3–5 questions evaluated self-perceived satisfaction toward the NPL intervention on a 5-point Likert scale (1 as the lowest and 5 as the highest). The questions on postsession feedback were (1) “Did you think the learning content of cerebrovascular diseases were appropriately arranged?” (2) “Did you enjoy NPL in standardized residency training?” (3) “Would you say that NPL helped you learn identified knowledge?” The questions on postprogram feedback were (1) “Did you find yourself helpful as a tutor?” (2) “Did you enjoy teaching in your NPL session?” (3) “Did you become more knowledgeable about the topic after the NPL session?” (4) “Did you feel NPL have helped in improving your teaching skills?” (5) “Did you think the NPL have helped in applying knowledge in clinical situations?” Teaching assistants and instructors were interviewed about their feedback on NPL after all sessions were completed (eAppendix 1, links.lww.com/NE9/A24).

### Statistical Analysis

Data were analyzed using SPSS version 22.0 (IBM Corp., Armonk, NY). Descriptive statistics were performed using mean with SD or median with interquartile range for continuous variables. A paired *t* test was used for parametric data and Wilcoxon signed-rank test for nonparametric data to compare scores on precourse/postcourse examinations. One-way univariate analysis of covariance adjusted for precourse scores was conducted to compare the postcourse scores between tutors and tutees. All statistical tests were 2-sided and considered significant at α < 0.05.

### Standard Protocol Approvals, Registrations, and Patient Consents

The ethical review board of PUMCH approved this study (I-22PJ273) because education research did not require informed consent.

### Data Availability

Data are available on reasonable requests to the authors.

## Results and Assessment Data

### General Information of Participation

From December 2019 to March 2022, the NPL-integrated neurology residency program was delivered annually over 3 academic years (Table 1). The numbers of residents in our residency training program were 18, 18, and 21, respectively (57 in total). All trainees were required to participate in NPL as a part of the training program requirements. Some participated in more than 1 academic year. Residents could be assigned as tutor or tutee in different years. Eighteen lectures were delivered by 15 tutors. Thirty-nine tutees attended the lectures.

**Table 1:** Number of Participants in Near-Peer Learning

Time	Year of residency	Tutee (N)	Tutor (N)	Total (N)
2019				18
	First year	4	1	5
	Second year	5	3	8
	Third year	3	2	5
2020				18
	First year	6	0	6
	Second year	5	0	5
	Third year	1	6	7
2021				21
	First year	9	0	9
	Second year	6	0	6
	Third year	0	6	6

### Assessments

#### Exams

All residents (including tutors and tutees) completed the precourse and postcourse examinations (Table 2). Scores on postcourse examinations were significantly higher than precourse scores (64.22 ± 12.11 vs 59.80 ± 15.88, *p* = 0.003). The largest improvement in scores was observed for first-time participants (58.26 ± 11.57 vs 51.75 ± 14.77, *p* = 0.001) and first-year residents (56.90 ± 13.10 vs 48.20 ± 15.55, *p* = 0.005). No difference was observed in the improvement in examination scores for tutors compared with tutees (*F* = 0.834, *p* = 0.365, by one-way univariate analysis).

**Table 2:** Precourse and Postcourse Examination Scores

	Frequencies of participation	Precourse Mean ± SD	Postcourse Mean ± SD	*Z*	*p* Value
Role					
Tutors	18	67.05 ± 14.91	69.89 ± 9.37	0.85	0.85
Tutees	39	56.45 ± 15.35	61.60 ± 12.43	2.93	0.003
Year of residency					
First year	20	48.20 ± 15.55	56.90 ± 13.10	2.84	0.005
Second year	20	63.30 ± 13.31	66.25 ± 10.45	1.49	0.14
Third year	17	69.33 ± 10.23	70.44 ± 8.16	0.4	0.69
Times of participation					
One time	34	51.75 ± 14.77	58.26 ± 11.57	3.22	0.001
Two times	18	70.19 ± 6.81	72.06 ± 6.41	0.89	0.37
Three times	5	77.10 ± 11.04	76.50 ± 2.60	0.13	0.89
Total	57	59.80 ± 15.88	64.22 ± 12.11	2.99	0.003

#### Feedback

Over the 3 academic years, 162 postsession feedback surveys were collected from participants immediately after each session and 15 postprogram surveys were collected from tutors up to 1–3 years after teaching (Table 3).

**Table 3:** Postsession Feedbacks From Participants

	Strongly disagree	Disagree	Not sure	Agree	Strongly agree
Did you think the learning content of cerebrovascular disease were appropriately arranged?	0	0	1%	10%	90%
Did you enjoy NPL in standardized residency training?	0	1%	1%	6%	92%
Would you say that NPL helped you learn identified knowledge?	0	0	0	13%	87%

Abbreviation: NPL = near-peer learning.

Regarding postsession feedbacks from participants, over 98% participants reported satisfaction toward the NPL intervention (Table 3). They agreed or strongly agreed that they enjoyed NPL in residency training and that NPL was helpful in knowledge acquisition. For future courses, participants suggest that more case discussions should be considered. Given the extensive course content, they also request prolonging the length of each lecture for sufficient time to achieve understanding. In addition, they specified a need for longer time on the principles and interpretation of TCD.

For postprogram feedback from tutors, the long-term effect of NPL was favorable. It was described as helpful, enjoyable, and knowledgeable (Table 4). Most tutors felt they were helpful and became more knowledgeable. They reported improvement in their teaching abilities and application of knowledge in clinical situations. As for their suggestions for further improvements for teaching, they reported that it was essential to have feedback on teaching performance from tutees, teaching assistants, and instructors. They appreciated that mistakes in theoretical knowledge and case-based discussions were pointed out explicitly by the instructors. In addition, they also indicated that course preparation was relatively time-consuming.

**Table 4:** Postprogram Feedbacks From Tutors

	Strongly disagree	Disagree	Not sure	Agree	Strongly agree
Did you find yourself helpful as a tutor?	7%	0	0	40%	53%
Did you enjoy teaching in your NPL session?	0	0	27%	27%	47%
Did you become more knowledgeable about the topic after NPL session?	0	13%	0	13%	73%
Did you feel NPL have helped in improving your teaching skills?	0	0	13%	27%	60%
Did you think NPL have helped in applying knowledge in clinical situations?	7%	7%	0	33%	53%

Abbreviation: NPL = near-peer learning.

As for the teaching assistants, the NPL consolidated their foundational knowledge of cerebrovascular diseases, which could contribute to leadership in the future.

From the instructors' perspectives, the NPL lessened their workload and improved teaching efficiency by explicitly demonstrating the mistakes of the residents.

## Discussion and Lessons Learned

In this study, we report our experience with implementing NPL in neurology residency training in a teaching hospital in China. Knowledge of the presentation and management of patients with cerebrovascular disease improved and was the greatest for first-time participants in NPL. Feedback on the educational value of NPL was favorable, with participants indicating NPL intervention to be enjoyable and helpful in gaining knowledge. These data suggest that NPL is a feasible instructional method and help residents acquire foundational knowledge of cerebrovascular diseases and provide the senior residents with teaching opportunities.

Improvements in examination scores and favorable learner feedbacks in this study suggest that NPL could assist residents in comprehending foundational knowledge of cerebrovascular diseases. Similar to previous studies, growing evidence had been established to support its implementation within medical curricula.^4,14-16^ The integrated theoretical model of peer learning proposed by Topping suggest that the effectiveness of peer learning lies in the processes of “organization and engagement, cognitive conflict, scaffolding and error management, communication, and affect.”^17^ Each of these was incorporated into our NPL intervention. Organization and engagement included several features of learning interactions that occurred in our course such as time engaged with tasks as well as collaboration between the tutor and the tutee. The learning process reported by tutees and tutors involved cognitive conflicts and challenges. Near-peers can help learners manage misconceptions and errors by thinking from a beginner's perspective. This creates an environment of psychological safety within the learning environment and could allow students to learn from errors more effectively. When tutors are trying to explain a concept to tutees, they have to grasp the concept comprehensively and improve their communication skills. In addition, participants felt psychologically safe and were more proactive in learning among peers. These processes promote both the tutors and tutees to learn in an NPL environment.

It was interesting that the first-year residents and first-time participants obtained the most remarkable improvements in the examination score. However, the improvements did not persist but were on a plateau. For one thing, this might indicate long-term knowledge retention after NPL, in line with previous literature.^14^ For another, it also implied that it might be unnecessary to require second-year residents participate in NPL again. That could save much time and avoid burn out for residents.

Previous studies demonstrate that tutors might obtain more improvements than tutees.^18^ However, this is not the case in our study. The underlying reasons might be the following: (1) These results might be underpowered because of small sample size and (2) the precourse examination was arranged 30 minutes before the first session when the tutors had already prepared the teaching materials. It is reasonable to assume that greater improvements in tutors might be achieved if the precourse examination was conducted before course preparation. Such arrangements were infeasible because of restrictions during the coronavirus disease 2019 pandemic in the past 3 years. Limited numbers of face-to-face classes were allowed.

We also gained several insights from feedback. First, foundational knowledge is more suitable for the near-peers to comprehend and teach. Nearly all participants reported satisfaction toward the arrangement of foundational knowledge of cerebrovascular disease. Near-peers were able to lead these lectures and case discussions. For more advanced knowledge such as reperfusion therapy in ischemic stroke and stroke in young adults, misconceptions can occur and confuse the tutees. Second, the NPL intervention is enjoyable for both tutors and tutees. We observed more relaxed atmosphere and engagement in NPL. Participants even asked for longer course length and more case-based discussions. Third, instructors reported that their burden was relieved during the NPL intervention when foundational knowledge was delivered by senior residents. This implies that NPL could be a win-win solution in areas with insufficient faculties.

In future iterations, several revisions could be made to address these recognized challenges with NPL. First, as for the teaching contents, more difficult materials, such as the principles and interpretation of TCD, might demand more time and illustration. Second, more case-based discussions on ischemic and hemorrhagic stroke could be applied. This would enhance residents' confidence and independency in clinical practice. Third, to relieve the burden of tutors when preparing for the course, recommended materials and teaching skills should be provided. Fourth, participation of second-year residents would not be required because their improvements in the postcourse/precourse examination score were not remarkable.

The strengths of NPL in this study included: (1) It was integrated into the neurology residency training on the foundational knowledge of cerebrovascular diseases. The anatomy of cerebral blood vessels and neuroimaging were difficult to memorize and easily forgotten. NPL might promote learner autonomy and engagement, leading to long-term knowledge retention. (2) The guidance of instructors specialized in cerebrovascular diseases with years of clinical practice and teaching experience assured the teaching quality. (3) Precourse/postcourse examinations as well as postsession and postprogram feedbacks were applied to assess the effectiveness and to allow for further revisions.

There are some limitations in this study. First, there was no comparator group in which the teaching was performed by faculty; therefore, the effectiveness of NPL compared with faculty-led teaching might be unknown. However, the number of neurology residents in our training base was too small even after we enrolled all residents in the program (18–21 people per year). Therefore, we applied the historical comparison among residents. Future studies might include more training centers to strengthen the efficacy of NPL in neurology residency training. Second, the reliability and difficulty level of home-grown examinations were uncertain. To lessen the bias, the questions were chosen from our neurology examination database with similar difficulty levels. They were further revised and approved by instructors. Third, the effectiveness of NPL in enhancing teaching competency was not sufficiently evaluated in this study. The evaluation of teaching ability was multifaceted and might manifest as a long-term effect. We obtained postprogram feedbacks from tutors 1–3 years after teaching. Future studies could involve assessments from tutors, colleagues, fellows, and instructors. Fourth, some residents participated in NPL more than once, which might affect the precourse examination scores.

Our study suggested that NPL might be feasible and enabled residents to gain foundational knowledge of cerebrovascular diseases. It also provided the senior residents with teaching opportunities. With some revisions, we intend to continue this NPL integrated residency training program annually. Future studies might explore its utilization in other residency programs in China.

## Appendix Authors

**Table:**

Name	Location	Contribution
Yuehui Hong, MD	Department of Neurology, State Key Laboratory of Complex Severe and Rare Diseases, Peking Union Medical College Hospital, Chinese Academy of Medical Science & Peking Union Medical College, Beijing, China	Drafting/revision of the manuscript for content, including medical writing for content; major role in the acquisition of data; study concept or design; analysis or interpretation of data
Ning Su, MD	Department of Neurology, State Key Laboratory of Complex Severe and Rare Diseases, Peking Union Medical College Hospital, Chinese Academy of Medical Science & Peking Union Medical College, Beijing, China	Drafting/revision of the manuscript for content, including medical writing for content; analysis or interpretation of data
Hanhui Fu, MD	Department of Neurology, State Key Laboratory of Complex Severe and Rare Diseases, Peking Union Medical College Hospital, Chinese Academy of Medical Science & Peking Union Medical College, Beijing, China	Drafting/revision of the manuscript for content, including medical writing for content; analysis or interpretation of data
Yuze Cao, MD	Department of Neurology, State Key Laboratory of Complex Severe and Rare Diseases, Peking Union Medical College Hospital, Chinese Academy of Medical Science & Peking Union Medical College, Beijing, China	Drafting/revision of the manuscript for content, including medical writing for content; analysis or interpretation of data
Ming Yao, MD	Department of Neurology, State Key Laboratory of Complex Severe and Rare Diseases, Peking Union Medical College Hospital, Chinese Academy of Medical Science & Peking Union Medical College, Beijing, China	Drafting/revision of the manuscript for content, including medical writing for content; analysis or interpretation of data
Lixin Zhou, MD	Department of Neurology, State Key Laboratory of Complex Severe and Rare Diseases, Peking Union Medical College Hospital, Chinese Academy of Medical Science & Peking Union Medical College, Beijing, China	Drafting/revision of the manuscript for content, including medical writing for content; analysis or interpretation of data
Jun Ni, MD, PhD	Department of Neurology, State Key Laboratory of Complex Severe and Rare Diseases, Peking Union Medical College Hospital, Chinese Academy of Medical Science & Peking Union Medical College, Beijing, China	Drafting/revision of the manuscript for content, including medical writing for content; study concept or design; analysis or interpretation of data
Yicheng Zhu, MD, PhD	Department of Neurology, State Key Laboratory of Complex Severe and Rare Diseases, Peking Union Medical College Hospital, Chinese Academy of Medical Science & Peking Union Medical College, Beijing, China	Drafting/revision of the manuscript for content, including medical writing for content; study concept or design

## Glossary

NPL: near-peer learning
PUMCH: Peking Union Medical College Hospital
SRT: standardized residency training
TCD: transcranial Doppler

## References

[1] Bega D, Krainc D. Challenges to neurology residency education in today's health care environment. Ann Neurol. 2016;80(3):315-320. doi:10.1002/ana.24729 27422739

[2] Zhu J, Li W, Chen L. Doctors in China: improving quality through modernisation of residency education. Lancet. 2016;388(10054):1922-1929. doi:10.1016/s0140-6736(16)00582-1 27339756

[3] Lio J, Dong H, Ye Y, Cooper B, Reddy S, Sherer R. Standardized residency programs in China: perspectives on training quality. Int J Med Educ. 2016;7:220-221. doi:10.5116/ijme.5780.9b85 27421072 PMC4958345

[4] Xu R, Duan C, He Q, et al. An observation of the peer-assisted learning (PAL) method in the clinical teaching of vertigo/dizziness-related diseases for standardized residency training (SRT) students in China: a randomized, controlled, multicenter study. BMC Med Educ. 2021;21(1):532. doi:10.1186/s12909-021-02969-1 34649532 PMC8518317

[5] Bulte C, Betts A, Garner K, Durning S. Student teaching: views of student near-peer teachers and learners. Med Teach. 2007;29(6):583-590. doi:10.1080/01421590701583824 17922356

[6] Ologunde R, Rabiu R. Students as teachers: the value of peer-led teaching. Perspect Med Educ. 2014;3(1):61-62. doi:10.1007/s40037-013-0094-8 24288126 PMC3889992

[7] Ransohoff A, Boscardin C, Hauer KE, Wlodarczyk S. Rethinking how to introduce the learning sciences: a near-peer approach. Med Sci Educ. 2021;31(1):45-47. doi:10.1007/s40670-020-01142-5 34457862 PMC8368513

[8] MacDonald M, Thompson AE, Ton J, Mysak T. Strategies to optimize implementation of novel preceptorship models: peer-assisted learning and near-peer teaching. Curr Pharm Teach Learn. 2020;12(8):945-955. doi:10.1016/j.cptl.2020.04.001 32564997

[9] Wu J, Jia J. Neurology, 3rd ed. People's Medical Publishing House; 2016.

[10] Peter Duus MB. Duus' Topical Diagnosis in Neurology: Anatomy, Physiology, Signs, Symptoms. Geory Thieme Verlag; 2006.

[11] Reif Ma Pocket Atlas of Sectional Anatomy, 4th ed. Thieme Medical Publishers; 2013.

[12] Alberstone CD. Anatomic Basis of Neurologic Diagnosis, 1st ed. Thieme Medical Publishers; 2009.

[13] Caplan LR. Caplan's Stroke: A Clinical Approach, 4th ed. Elsevier and Peking University Medical Press; 2010.

[14] Agius A, Stabile I. Undergraduate peer assisted learning tutors' performance in summative anatomy examinations: a pilot study. Int J Med Educ. 2018;9:93-98. doi:10.5116/ijme.5aa3.e2a6 29621744 PMC5951778

[15] Sethi S, Dabas R, Garg R. Near-peer-assisted learning for training undergraduate medical students in clinical ophthalmology skills. J Taibah Univ Med Sci. 2022;17(1):105-111. doi:10.1016/j.jtumed.2021.10.008 35140572 PMC8801524

[16] Musbahi A, Sharpe A, Straughan R, Ong S, Alhaddabi A, Reddy A. A near-peer regional surgical teaching programme designed by medical students, delivered by junior doctors. Med Educ Online. 2019;24(1):1583969. doi:10.1080/10872981.2019.1583969 30924405 PMC6442097

[17] Topping KJ. Trends in peer learning. Educ Psychol. 2005;25(6):631-645. doi:10.1080/01443410500345172

[18] Williams B, Reddy P. Does peer-assisted learning improve academic performance? A scoping review. Nurse Educ Today. 2016;42:23-29. doi:10.1016/j.nedt.2016.03.024 27237348

